# Idiopathic Dendriform Pulmonary Ossification Diagnosed by Transbronchial Lung Cryobiopsy: A Case Report

**DOI:** 10.7759/cureus.77955

**Published:** 2025-01-25

**Authors:** Masamichi Komatsu, Fumika Sugiyama, Keisuke Todoroki, Momoko Takizawa, Masayuki Hanaoka

**Affiliations:** 1 First Department of Internal Medicine, Shinshu University School of Medicine, Matsumoto, JPN; 2 Department of Radiology, Shinshu University School of Medicine, Matsumoto, JPN; 3 Department of Laboratory Medicine, Shinshu University School of Medicine, Matsumoto, JPN

**Keywords:** dendriform pulmonary ossification, idiopathic dendriform pulmonary ossification, interstitial lung abnormalities, pulmonary ossification, transbronchial lung cryobiopsy

## Abstract

Dendriform pulmonary ossification (DPO) is a rare condition characterized by ossification in the lungs. Lung ossification can be categorized into idiopathic ossification or secondary ossification associated with underlying pulmonary, cardiac, and systemic disorders. We herein present a case of a 60-year-old man with bilateral reticulonodular shadows detected on chest radiography during a medical checkup. A transbronchial lung biopsy did not provide a definitive diagnosis, and the asymptomatic patient was referred to our hospital due to worsening imaging findings. Further evaluation, including transbronchial lung cryobiopsy (TBLC), revealed dendriform ossification with components of bone marrow within the lung parenchyma. In the absence of underlying disease, the patient was diagnosed with idiopathic DPO (IDPO). This case highlights the importance of considering DPO in the differential diagnosis of unexplained pulmonary ossification and suggests that TBLC is a useful diagnostic tool for IDPO.

## Introduction

In cases where pulmonary ossification is observed on chest computed tomography (CT), both primary and secondary dendriform pulmonary ossification (DPO) should be considered [1]. Secondary causes, such as interstitial pneumonia or heart failure, should be explored as the potential origin of DPO [1]. While secondary pulmonary ossification is the more common presentation of DPO, idiopathic DPO (IDPO) is relatively rare [2,3]. Traditionally, the clinical course of IDPO has been considered asymptomatic and slow-progressing [3]. However, recent studies suggest that IDPO may progress over the long term [4]. Therefore, obtaining a histological diagnosis is crucial.

Conventional transbronchial lung biopsy (TBLB) can only collect small tissues, making DPO difficult to diagnose. In recent years, transbronchial lung cryobiopsy (TBLC), a new diagnostic procedure has emerged, making collection of larger tissues possible. Herein, we present a case of IDPO diagnosed via TBLC.

## Case presentation

A 60-year-old man presented to an initial unaffiliated hospital with bilateral reticulonodular shadows on chest radiography, which were observed during a medical check-up five years ago. Four years prior to presentation, a TBLB was performed at the same hospital, but it did not yield a definitive diagnosis. The patient was asymptomatic, but worsening imaging findings prompted a referral to our hospital for further evaluation. His medical history included obstructive sleep apnea that was well-managed with continuous positive airway pressure. He was a former smoker of 10 cigarettes per day for 20 years. There was no family history of lung disease. He worked in an office environment with no known dust exposure.

**Figure 1:**
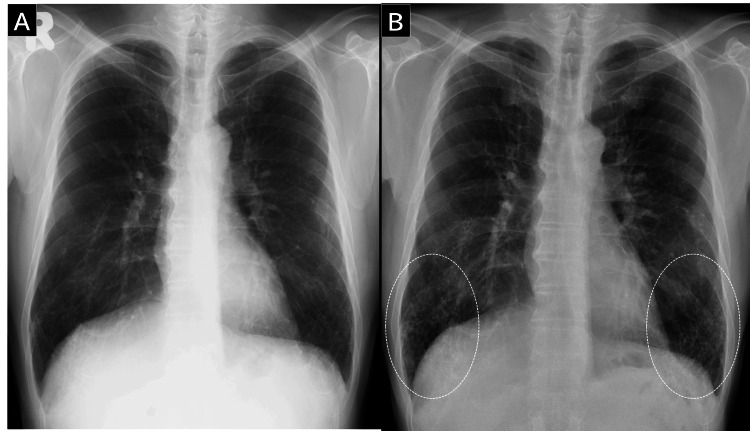
Chest radiography findings over time (A) Chest radiograph at medical check-up. (B) The bilateral reticulonodular shadows in the lower lung zone (circle) became clearer after five years.

**Figure 2:**
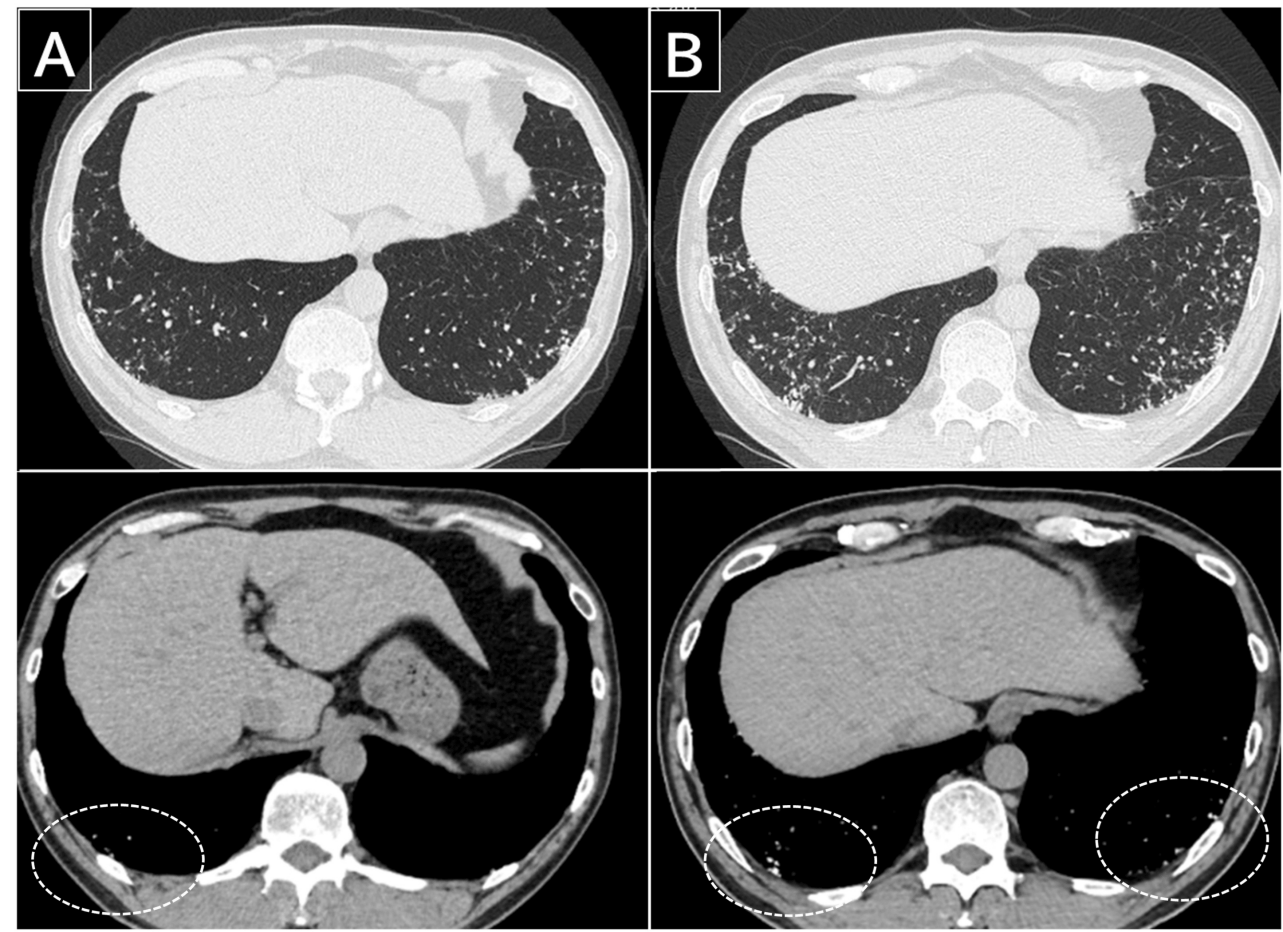
Chest computed tomography findings over time (A) Bilateral, diffuse, linear, and branching structures with calcification predominantly in the lower lung lobes (circle). (B) Progression of ossified lesions over a five-year period.

Upon examination, the patient’s vital signs were normal. Physical examination revealed no chest abnormalities or signs suggestive of collagen vascular disease. His blood and serum chemistry test results were within normal limits. Pulmonary function test results were normal: vital capacity of 4.85 L (105.4%), forced vital capacity of 4.86 L (109%), forced expiratory volume in 1 s of 3.52 L (94.4%), and diffusing capacity of the lung for carbon monoxide of 23.41 mL/min/mmHg (104%). Chest radiography showed deterioration over the five-year period, with bilateral reticulonodular shadows evident in the lower lung zone (Figure 1). Chest CT revealed bilateral, diffuse, linear, and branching structures with calcification, predominantly in the lower lung lobes (Figure 2).

**Figure 3:**
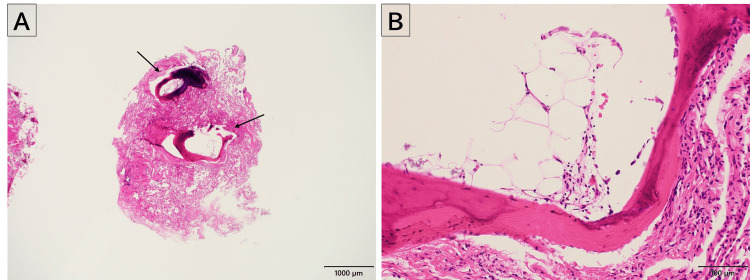
Pathological findings from transbronchial lung cryobiopsy Pathological findings from transbronchial lung cryobiopsy showing dendriform ossification (arrow) with mature bone marrow within the lung parenchyma. Hematoxylin and eosin staining at low magnification (×20, A) and high magnification (×200, B).

Based on these findings, a TBLC from the right lower lobe was performed. Histopathological examination revealed dendriform ossification with a component of the bone marrow within the lung parenchyma (Figure 3).

There were no findings suggestive of secondary pulmonary ossification associated with heart failure, fibrotic interstitial pneumonia, or emphysema. Consequently, the patient was diagnosed with IDPO after a multidisciplinary discussion. Given the absence of symptoms and normal pulmonary function tests, the patient was managed with regular follow-up, and no immediate treatment was initiated.

## Discussion

IDPO is a rare condition characterized by diffuse ossification in the lung parenchyma. While secondary ossification associated with heart failure, fibrotic interstitial pneumonia, emphysema, pneumoconiosis, or aspiration pneumonia is relatively common, IDPO is considered extremely rare [2,3,5]. Table 1 shows the differential diagnosis when pulmonary ossification is observed [1]. The pathogenesis of pulmonary ossification is unknown, but it is believed to involve multiple factors, such as tissue injury, precipitation of calcium salts, and activate profibrogenic cytokines [6].

**Table 1:** Differential diagnosis of pulmonary ossification

Differential diagnosis
Idiopathic pulmonary ossification
Preexisting pulmonary disorder
Idiopathic pulmonary fibrosis
Pulmonary amyloidosis
Acute respiratory distress syndrome
Sarcoidosis
Tuberculosis
Metastatic cancer
Preexisting cardiac disorder
Mitral stenosis
Chronic heart failure
Preexisting extracardiopulmonary disorder
Primary and secondary hyperparathyroidism
Hypervitaminosis D

Historically, IDPO was regarded as a condition with minimal symptoms and slow progression. A recent comprehensive nationwide retrospective study from Japan [4] revealed that the average age of onset for IDPO is relatively low, at 37 years, with 82% of cases occurring in nonsmokers. While most cases remain asymptomatic, 36% exhibit impaired pulmonary function at the time of diagnosis. Long-term follow-up chest CTs have shown progression of ossification lesions in most cases, with no cases demonstrating spontaneous regression. Approximately 30% of cases showed a decline in pulmonary function, thereby meeting the criteria for progressive fibrosing interstitial lung diseases [7].

Interstitial lung abnormalities (ILAs) are incidental findings on chest CT, characterized by non-dependent abnormalities in the lungs [8]. ILAs are associated with respiratory symptoms [9], decreased exercise capacity [10], reduced pulmonary function [11], and increased mortality [12]. Radiographic findings of lung calcifications account for 9% of ILAs [13]. In this case, the patient was referred to the previous hospital due to an abnormal shadow detected during a medical check-up.

Histological examination is crucial for diagnosing IDPO owing to its progressive nature. While surgical lung biopsy (SLB) is useful for diagnosis [14,15], it may be avoided in asymptomatic cases of IDPO. Recently, TBLC has emerged as an alternative to SLB [16]. Notably, Sekimoto et al. reported a case of IDPO diagnosed by TBLC [17]. TBLC is less invasive than SLB [18] and allows for the collection of larger specimens than TBLB. Therefore, TBLC may be a promising method for diagnosing IDPO. Increased histological examination of IDPO may provide insights into the pathogenesis of pulmonary ossification and its underlying genetic factors.

## Conclusions

In this report, we described the case of a patient with IDPO, which was diagnosed using TBLC. Some cases of IDPO are progressive, making histological diagnosis crucial. TBLC is a less invasive and effective method for diagnosing IDPO.
